# Dueling biological and social contagions

**DOI:** 10.1038/srep43634

**Published:** 2017-03-02

**Authors:** Feng Fu, Nicholas A. Christakis, James H. Fowler

**Affiliations:** 1 Department of Mathematics, Dartmouth College, Hanover, NH 03755, USA; 2 Department of Biomedical Data Science, Geisel School of Medicine at Dartmouth, Lebanon, NH 03756, USA; 3 Department of Medicine, Yale University, New Haven, Connecticut 06520, USA; 4 Department of Sociology, Yale University, New Haven, Connecticut 06520, USA; 5 Yale Institute of Network Science, Yale University, PO Box 208263, New Haven, Connecticut 06520, USA; 6 Division of Global Public Health, University of California, San Diego, La Jolla, CA 92093, USA; 7 Department of Political Science, University of California, San Diego, La Jolla, CA 92093, USA

## Abstract

Numerous models explore how a wide variety of biological and social phenomena spread in social networks. However, these models implicitly assume that the spread of one phenomenon is not affected by the spread of another. Here, we develop a model of “dueling contagions”, with a particular illustration of a situation where one is biological (influenza) and the other is social (flu vaccination). We apply the model to unique time series data collected during the 2009 H1N1 epidemic that includes information about vaccination, flu, and face-to-face social networks. The results show that well-connected individuals are more likely to get vaccinated, as are people who are exposed to friends who get vaccinated or are exposed to friends who get the flu. Our dueling contagion model suggests that other epidemiological models may be dramatically underestimating the *R*_0_ of contagions. It also suggests that the rate of vaccination contagion may be even more important than the biological contagion in determining the course of the disease. These results suggest that real world and online platforms that make it easier to see when friends have been vaccinated (personalized vaccination campaigns) and when they get the flu (personalized flu warnings) could have a large impact on reducing the severity of epidemics. They also suggest possible benefits from understanding the coevolution of many kinds of dueling contagions.

Voluntary mass vaccination is a fundamental strategy to achieve ‘herd immunity’ and to limit vaccine-preventable contagions^1^,^2^,^3^,^4^,^5^. However, a misalignment between individual self-interest and the public interest causes many people to remain unvaccinated^6^,^7^,^8^,^9^, which can expose the population to significant disease outbreaks and compromise efforts to eradicate the diseases in question. To understand vaccine compliance, considerable attention has been focused on integrating epidemiology with game-theoretic behavior models in recent years^10^,^11^,^12^,^13^,^14^,^15^,^16^,^17^,^18^,^19^,^20^,^21^,^22^,^23^. And while these models have devoted considerable attention to the incentives facing individuals deciding whether or not to vaccinate given the threat posed by the contagion in question, they have paid less attention to the social effect of those decisions on others.

Yet, accumulating empirical data suggests that the phenomenon of social contagion is common^24^,^25^,^26^, especially when it comes to health decisions, such as smoking cessation^27^ and vaccine adoption^28^,^29^. Although a multitude of complex social factors may be involved^30^, the propagation of health behavior occurs when individual decisions are influenced by peers in social networks^10^,^12^,^14^,^15^,^18^,^21^,^22^,^31^. That is, social phenomena may spread interpersonally in a manner similar (though not necessarily identical)^26^,^31^ to biological contagion. And, in many public health settings, such social and biological contagions are often not independent^21^; rather, they may interfere with each other, and may even be seen as *dueling contagions*. Indeed, a feedback loop can arise between the spread of an epidemic and the spread of health behaviours^20^. In response to perceived risks and social influence, individuals may take preventative measures such as vaccination^28^ or reduced contact with others^13^, and these behavioural responses in turn modify the spread of infection^32^,^33^,^34^. It is, therefore, important to achieve a comprehensive understanding of the rich dynamics generated by this feedback process.

Here, we reconstructed the temporal dynamics of concurrent spreading of vaccination behaviour and seasonal influenza in a real social network (Fig. 1). The dataset has information regarding social network ties and individuals’ health status during the 2009 H1N1 flu epidemic^35^. We note that data of this kind are both very scarce and also particularly well suited for the study of dueling contagions. Although it is common for epidemiological studies to estimate key epidemiological parameters such as the basic reproductive ratio^36^ (denoted by *R*_0_), to our best knowledge, the dueling aspect of vaccination and infection has yet to be assessed using real data. For this reason, our focus is on inferring key characteristics of the dueling contagion processes, including how severe the epidemic really is (*R*_0_) and how promptly the focal population responds to the epidemic with vaccination.

Understanding the spread of either phenomenon (vaccination or infection) requires modeling the spread of both simultaneously. To quantitatively assess the degree of disease-behaviour interaction, we fit a simple dueling contagion model to the real data, showing that the vaccination decisions of individuals are jointly influenced by the prevalence of both the flu status and the vaccination status of their social contacts. Moreover, the modeling results suggest that the epidemic size strongly depends on how swiftly individuals respond with vaccination, and that it could be cut in half if the spread of vaccination behaviour was, say, twice as fast.

We enrolled a total of 744 undergraduate students from Harvard College, discerned their friendship ties, and tracked whether they had the flu beginning on September 1, 2009 (from the start of the new academic year) to December 31, 2009. This sample was assembled from two groups of students: (1) a sample chosen randomly from the 6,650 Harvard undergraduates (*N* = 319), and (2) a “friends” sample (*N* = 425) composed of individuals who were named as a friend at least once by a member of this random sample (see SI). The mapped social network consists of 750 pairs of directed friendships among the participants; there were 158 mutual ties and 592 pairs of unidirectional friendships (Fig. 1b–d). Such directionality of friendship ties does not necessarily imply the direction of the underlying peer influence or disease transmission; hence, in our analyses, we symmetrize the ties and convert the network into an undirected one; and the network degree of each subject is defined as the number of undirected friendships he/she has in this mapped social network. It is unlikely that friendship ties can vary meaningfully over short timescales such as in a 4-month window^37^; therefore, we assume that the friendship network of students in our sample did not change meaningfully over the period from September to December, and we treat the network as static over this time interval.

All subjects completed a brief background questionnaire soliciting demographic information. We obtained basic administrative data from the Harvard College registrar, such as sex and class of enrollment, and tracked cases of formally diagnosed influenza among the students in our sample as recorded by University Health Services (UHS) beginning on September 1, 2009 through December 31, 2009. We also collected biweekly self-reports, in order to complement the UHS vaccination records, to ascertain whether the students reported having been vaccinated (with seasonal flu vaccine or H1N1 vaccine or both) at places other than (and including) UHS. The total number of subjects who chose to be vaccinated (received flu shots) and/or got flu steadily increased during the study period and reached a plateau in late December. At the end of the flu season, 309 subjects had been vaccinated, and 57 had been diagnosed with the flu by clinicians.

## Results

**Figure 1:**
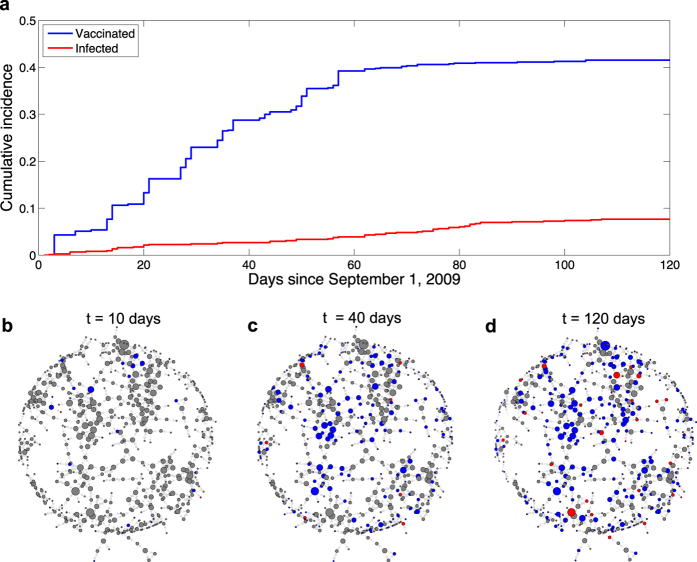
Temporal dynamics of concurrent spreading of vaccination and flu in a real social network. (**a**) Shows the cumulative incidence of vaccinated individuals and infected ones since September 1, 2009. The levels of vaccination coverage and of disease prevalence increase from zero and reach a plateau almost at the same time. These population aggregate behaviors offer a macroscopic view of the dueling contagions of vaccination and infection. (**b**–**d**) Display the snapshots of the social network at time point *t* = 10, 40, 120 days, respectively. Size of nodes is proportional to their network degree, and the color of nodes represents their health status: red infected, blue vaccinated and gray unvaccinated & healthy. These snapshots provide a microscopic, spatio-temporal view of the dueling contagions on the mapped friendship network, showing that the relatively fast spread of vaccination behavior impedes the development of a severe epidemic.

Figure 1a shows the temporal dynamics of vaccination (the blue line) and infection (the red line); both rose gradually and then subsequently entered saturation phases, although the spread of vaccination behaviour far outpaced the flu. As shown in the snapshots taken at different stages (Fig. 1b,c,d), we observe pronounced clustering of vaccinated individuals, whereas it appears that infected individuals did not form contiguous clusters and, in some cases, were isolated from the sampled population. This suggests a possible disease-behaviour interaction where fast spread of vaccination behaviour may have prevented the development of a severe flu epidemic.

**Figure 2:**
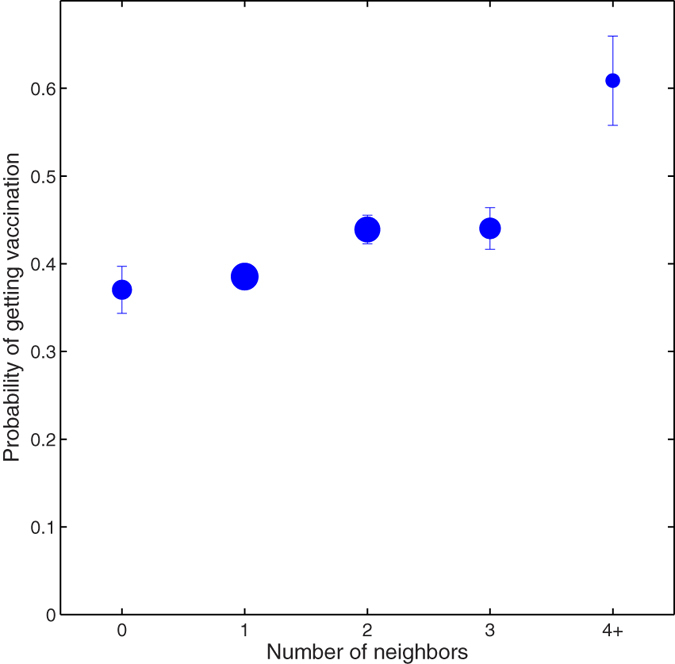
Social network degree as a determinant of vaccination. The plot shows the average probability of subjects getting vaccinated, grouped by their network degree. The error bars denote one standard deviation of the estimated mean. This empirical observation validates a previous theoretical prediction^14^: compared to periphery small-degree individuals, social hubs are more inclined to get vaccination possibly because of their increased chance of being exposed to others’ vaccination behavior as well as to the risk of infection.

In Fig. 2, we analyze how the probability of getting vaccinated depends on attributes of, and events in, one’s friends. First, it shows that the probability of choosing to be vaccinated is positively and significantly correlated with network degree (logistic regression coefficient = 0.17, *p* = 0.03). This result confirms a prior theoretical prediction^14^: since well-connected individuals have a greater chance of being exposed to the risk of infection, as well as to the vaccination behaviours of others, they should be more likely to choose to be vaccinated. In fact, there is some evidence for both of these mechanisms in our data. In the largest connected component of our sample network, for example, the vaccination rate (per individual per day) for individuals having at least one vaccinated friend is 0.0086 (95% CI, 0.0055–0.0132), much higher than that for individuals having 0 vaccinated friends, 0.0053, (95% CI, 0.0032–0.0087); and the vaccination rate for individuals having at least one friend who contracted the flu is 0.0078 (95% CI, 0.0020–0.0242), which is greater than that for individuals having 0 friends getting the flu, 0.0065 (95% CI, 0.0047–0.0091).

We now develop a simple model in order to understand the interplay between social and biological contagions. Consistent with previous studies^1^,^3^,^38^, we use the susceptible-infected-recovered model for the transmission of flu and a simple social contagion model for the spread of vaccination behaviour^34^. For generality, we consider dueling contagions on multiplex networks, where disease transmission does not necessarily follow the same pathways as social contagion. This is particularly important for our model because flu can spread via incidental contact, but behaviors are more likely to spread via strong social ties^39^. If *N* is the total number of subjects, then we can denote the *N* × *N* adjacency matrix of the social contagion network by 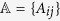, and that of the biological contagion network by 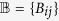. Let 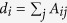 denote the number of neighbours of individual *i* in the social contagion network, and let 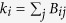 denote the number of neighbours of individual *i* in the biological contagion network.

Let 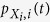 be the probability that individual *i* is in state *x*_*i*_ at time *t (X*_*i*_ = {*S, I, R, V*}). In the classic SIR model, *β* is the transmission rate of infection (*I*) and *γ* is the rate of recovery (*R*), and the well-known basic reproductive ratio (*R*_0_), which describes the severity of an epidemic, is simply *R*_0_ = *β*/*γ*. In our model, we add an additional state, vaccinated (*V*), and define a social contagion process that governs it. When individuals evaluate their vaccination choices, their decision-making may be driven by the vaccination behaviours of others through social influence^10^,^12^,^18^,^22^ and also by their assessment of infection risk through the level of flu prevalence around them. We use the parameter *a* ∈ (0, 1) to quantify the extent to which social contagion, as opposed to the current severity of the flu epidemic, plays a part in the focal individual’s vaccination decision. Since infection and vaccination decisions may occur on different time scales, we add an additional parameter, *ω*, to indicate the rate at which individuals make vaccination decisions. We also allow for a parameter *κ*, which indicates the maximum fraction of the population that will get vaccinated (the vaccination saturation level).

Each individual *i* has four possible states *S, I, R*, and *V*, and thus the dueling contagion process is a Markov chain on the space of 4^*N*^ states. The first order approximation of this Markov process can be described by the following ordinary differential equations:


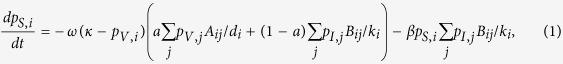



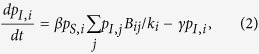



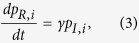



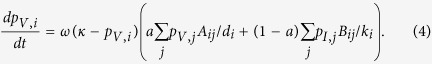


Here, we assume that the spread of flu is passive while the diffusion of vaccination is responsive, and that both processes take place along social interactions initiated by susceptible individuals. It is worth noting that this assumption differs from models that focus on the infectivity of disease spreaders who can actively infect their neighbors^40^,^41^. As all social interactions require a certain amount of time and effort and the number of interactions that each susceptible individual can initiate per unit time is limited, we normalize the interaction frequency of each focal susceptible individual *i* (i.e., one social interaction at a time). Specifically, the denominators 1/*k*_*i*_ and 1/*d*_*i*_ in the right hand side of eq. (1) account for the probability of interacting with one particular neighbor randomly chosen from *i*’s entire neighborhood. Moreover, by letting 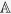 and 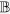 both be a matrix of ones, we obtain a coarse-grained version of dueling contagion version eqs (5–8) without the need of rescaling the model parameters with the population size *N*. This makes our model parameter estimates directly comparable between different scenarios (network-specific model vs. coarse-grained model).

**Figure 3:**
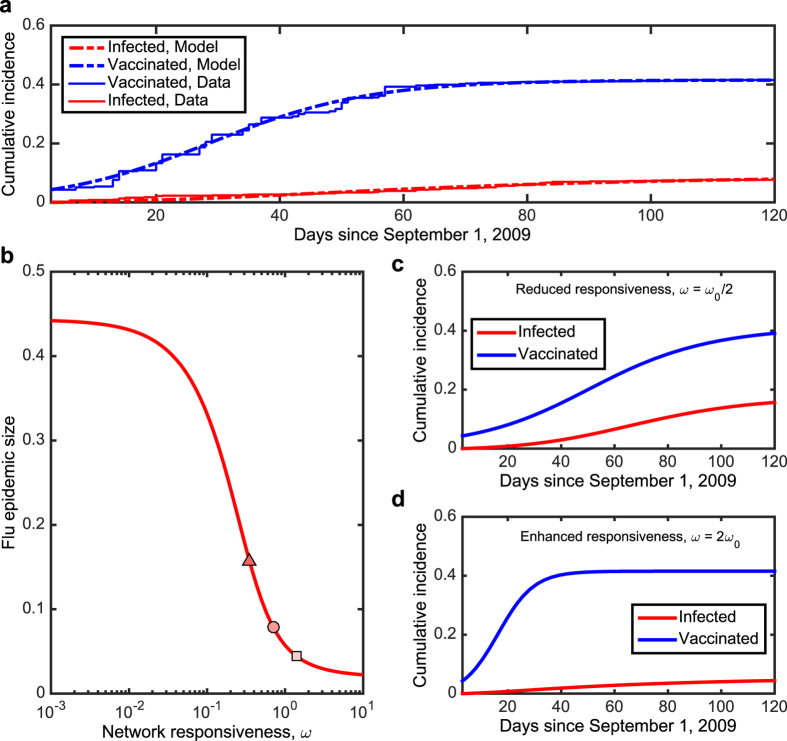
Modeling “dueling contagions”. (**a**) Shown are the real data regarding the population aggregate levels as in Fig. 1a (solid) and the best-fitting curves (dashed) using a simple dueling contagion model. Panel (b) shows the dependence of the epidemic size (*t* = 120) on the level of network responsiveness, *ω*, for other model parameters fixed with the estimated values. The circle marks the estimated value of *ω*_0_. (**c**) and (**d**) Plotted are the predictions of population aggregate behaviors, based on the mean-field approximation of the dueling contagion processes, for a smaller *ω* = *ω*_0_/2 (the triangle in panel b) and for a larger *ω* = 2*ω*_0_ (the square in panel b), respectively. Our dataset allows us to infer the time scales that govern the dueling contagions of vaccination and infection: the estimated *ω*_0_ ≈ 0.70 and *R*_0_ ≈ 1.56. Intermediate values of *ω* induce simultaneous interdependence between vaccination and infection. On the other hand, extreme values of *ω* lead to time-scale separation in which one contagion dynamic is much faster or slower than the other. The health outcomes could be further improved if individuals more promptly had themselves vaccinated through social influence and/or in response to the epidemic: the epidemic size could be mitigated almost by half if the spread of vaccinating behavior was twice as fast. Model fitting results and simulations are based on the corse-grained version of the dueling contagion model, eqs (5–8).

**Figure 4:**
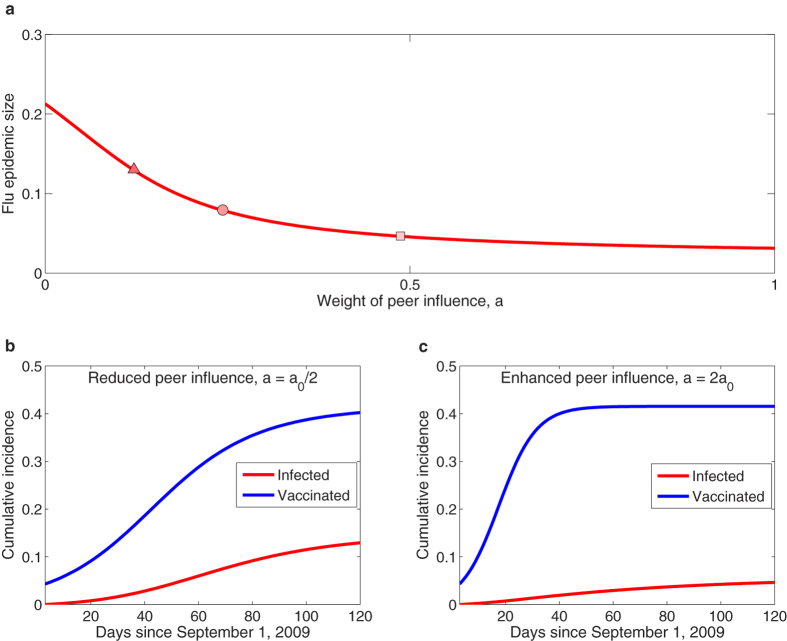
Positive consequences of social contagion on public health. Panel (a) depicts the final epidemic size (*t* = 120) as a function of the parameter *a* describing the extent of the role that social contagion, in comparison to the risk of infection, plays in an individual’s vaccination decision. The circle marks the estimated value of *a*_0_ ≈ 0.24 inferred from our real data. Panels (b) and (c) plot the population aggregate levels of vaccination and infection, predicted by our dueling contagion model, with halving (*a* = *a*_0_/2, the triangle in panel a) versus doubling (*a* = 2*a*_0_, the square in panel a) the relative effect of peer influence on vaccinating decisions of individuals. It seems rational for one to decide whether or not to be vaccinated according to the level of disease prevalence, but paradoxically the health outcome is worsened, as self-interest and social optimum are at odds in this case. In contrast, herd behavior, induced by social influence, rapidly boosts the uptake level and thus most improves the health outcome: the epidemic size could be reduced by half if the spread of vaccination behavior is driven only by social contagion (*a* = 1). Simulated results are based on the corse-grained version of the dueling contagion model, eqs (5–8), using the best estimated values of the model parameters.

In general, the Markov chain of this dueling contagion process is mathematically intractable. Using appropriate initial conditions, we can numerically solve these 4*N* equations (or more precisely, 3*N independent* equations due to the normalization conditions 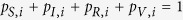 for 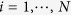) and get the expected equilibrium distribution of states for the entire population if it is small. For practical reasons, we choose to apply the network-specific dueling contagion model eqs (1–4) to a subset of our data (see the SI for more details). We assume that the adjacency matrices 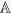 and 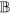 are symmetric (as previously noted) and are identical (both the biological contagion network and the social contagion network are represented by the mapped social network). We report the best parameter estimates in the last column of Table S1 in the SI. For comparison, we also plot similar figures corresponding to Figs 3 and 4 shown below (Figs S1 and S2 in the SI). We confirm that these results for the network-specific dueling contagion model are consistent with those reported here using the coarse-grained model.

For larger populations and those with only partially observed network data, it may be more appropriate to use approximations of contact with infected and vaccinated individuals based on population averages. Therefore, we also work with a coarse-grained version of the above equations where *ρ*_*X*_(*t*) is the fraction of the population in state 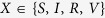 at time *t*:


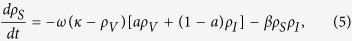



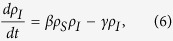



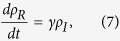



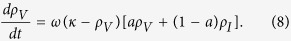


Following the common practice in modeling diffusion of innovation^42^, here we model the spread of vaccine adoption as a variant of logistic growth as given in eq. (8). Indeed, these vaccinated individuals were previously susceptible individuals (i.e., the first term in the right hand side of eq. (5) represents the rate of such state changes from *S* to *V* due to the spread of vaccine adoption), but not all remaining susceptible individuals that are spared from the infection will become vaccinated. Hence we use the term (*κ* − *ρ*_*V*_), rather than *ρ*_*S*_, to regulate the saturation effect for population adoption rates of vaccination.

We apply this model to our data, using simulated annealing to obtain estimates of the model parameters (see SI). Figure 3a shows a close fit between the model and the data, and estimates for all parameters are shown in the first column of Table 1. We compared the full model to several other variants, including a “rational response” model that assumes no behavioral contagion in vaccination (*a* = 0), a “social contagion” model that assumes vaccination decisions are not sensitive to the number of friends who have the flu (*a* = 1), and a “no vaccination” model that assumes no vaccination (see Table 1). After fitting each of these models to the data, we compared them using an Akaike criterion^18^,^22^,^43^. The best-fitting model was the full model, suggesting that vaccination decisions are important to the model and depend on both the infection rate and the behavioral contagion.

The model also suggests that other currently-used epidemiological models may be significantly underestimating *R*_0_, a key parameter used to assess the severity of a disease outbreak, if measures to contain the infection are being implemented in the population. In the best-fitting full model that fully accounts for equilibrium interactions between the dueling biological and social contagions, we estimate that *R*_0_ = 1.56. This contrasts with an estimate of *R* = 1.02 for the model that ignores vaccination altogether. Meanwhile, the model that allows for rational response but ignores social contagion errs on the opposite side, yielding an *R*_0_ = 1.77. These results suggest that it is critical to include social contagion of vaccination in disease models.

We used the best-fitting model to explore how each parameter influences the dueling contagion processes. The “responsiveness” parameter *ω* measures the rate at which individuals react to their friends’ vaccination behavior and infection status. Figure 3b shows that, for large *ω* values, the population reaches a high vaccination level quickly, thus providing sufficient herd immunity in time to reduce the incidence of flu to near zero. On the contrary, for small *ω* values, the vaccination level takes longer to reach the plateau due to slow response (as individuals ‘wait and see’ ref. 44), thus leading to unfavourable outcomes with large numbers of individuals infected.

However, the best-fitting *ω* is an intermediate value where small changes can have a large effect on the size of the epidemic. Halving the responsiveness causes the epidemic size to increase by 99% (Fig. 3c), while doubling responsiveness causes the epidemic to decrease by 44% (Fig. 3d). Importantly, these results suggest that efforts to alter the course of vaccination contagion by informing individuals about their friends’ decisions and outcomes (perhaps by using social media) could have a profound effect on the course of the disease.

The parameter *a* indicates how much individuals are influenced by the vaccination decisions of their friends relative to how much they are influenced by their friends’ flu status. The best-fitting estimate for this value is less than one half, suggesting that behavior in our sample is governed more by a response to friends who get the flu than by peer influence in vaccination. Nonetheless, Fig. 4a shows that the final epidemic size decreases asymptotically with increasing *a*. The reason for this is a difference in feedback mechanisms. Under peer influence, each vaccination increases the rate of vaccination in friends (positive feedback), but under “rational” response to flu status, each person who gets the flu causes more friends to get vaccinated, which decreases the rate of getting the flu (negative feedback). It is not obvious which of these two processes would dominate since vaccination decreases the incidence of flu, but the model suggests that the size of the epidemic is more sensitive to social contagion in vaccination than to the rate of flu in one’s peers. In fact, our model suggests that halving the relative effect of peer influence on vaccination decisions causes the epidemic size to increase by 64% (Fig. 4b), while doubling the effect of peers causes the epidemic to decrease by 41% (Fig. 4c).

## Discussion

Pre-emptive vaccination against epidemic diseases^4^,^45^ requires prompt actions by individuals so as to achieve both personal and herd immunity swiftly, thus mitigating the impact of epidemic outbreaks. Past work has explored disease-behaviour interactions^8^,^10^,^11^,^14^,^19^, but the model we present here is the first, we believe, to consider how the spread of behavior coevolves with the spread of disease, using real data to investigate key aspects of these dueling contagions. Among the most important results, we find that personal vaccination decisions tend to be influenced by friends who get the flu or who themselves decide to get vaccinated. A failure to account for this dependency can substantially alter the most basic measure of the severity of an epidemic, *R*_0_. The social effect of flu and vaccination on others’ vaccination decisions can have a profound effect on the final size of an epidemic, and, while the model suggests that people in our sample are more affected when their friends get the flu, the size of epidemics is much more sensitive to the extent to which people copy one another’s vaccination behavior.

These results suggest that organizations charged with monitoring and predicting the course of disease should incorporate behavioral contagion into their models. They should also explore interventions that increase the visibility of friend outcomes and behaviors to exploit peer effects for the mitigation of epidemics. For example, an online application that lets people know when one of their friends has the flu (personalized flu warnings) may spur more people to get vaccinated, and, even better, an application that lets people know when their friends get vaccinated (personalized vaccination campaigns) could have a huge effect on increasing the rate of vaccination (as seen with voting notification)^46^. Even without technology, a campaign to ask people to tell their friends when they get vaccinated or have the flu could also be effective. The key is to use social contagions to fight biological contagions, helping the former to outstrip the latter.

Another implication of this work is that being well-connected to the social network can, paradoxically, actually be advantageous under certain circumstances. Central individuals not only perceive earlier warning signs regarding biological contagion^35^ – they are also more likely to be exposed to friends who have gotten vaccinated. Both of these tendencies increase the likelihood that central individuals will get vaccinated sooner, and, if the social contagion outpaces the biological contagion, it could paradoxically reduce the rate of flu in central individuals compared to those at the periphery of the network. This is an important insight that warrants further theoretical investigation, as central individuals are ordinarily seen as having an increased risk of infection during contagious outbreaks.

We have investigated the spread of a positive health behavior here, but we emphasize that social contagions may also promote the spread of misinformation and bad health behaviors, as well^29^,^31^. The spread of vaccine avoidance (due to misinformation) among parents (via social contagion) has caused the vaccination rates of newborns in many parts of the USA to plunge from high levels, which in turn has increased the incidence of several childhood diseases (via biological contagion)^10^,^18^. These fears are fueled not only by face-to-face interaction, but also by changing opinions of vaccination that are expressed in online social media^29^. It is critical for us to better understand these spreading processes so that public health efforts can take advantage of the positive effects of social contagion, while ameliorating their potential negative impacts.

It remains empirically quite difficult to gather reliable and detailed information on both biological and social contagion networks and the structures of multiplex networks in general^47^. For this reason, the current dataset only permits us to obtain reliable parameter estimates of dueling contagion processes using a population model instead of the network-specific one. This first approximation, while enlightening, may underestimate some of the spreading dynamics that would occur in more realistically observed networks, especially when taking into account the heterogeneity in the biological contagion network^40^,^41^,^48^. Nevertheless, in cases where it becomes feasible to obtain a high-resolution biological and social network data, perhaps using “big data” techniques, the dueling contagion model as introduced here can be tailored to explicitly take such realistic, multiplex networks into account. Doing so will certainly help improve the quantitative assessment of the disease-behaviour interaction and thus will help us to make better predictions relevant for public health policy.

Failures of vaccination to protect against flu^38^, although uncommon, poses a second order problem^49^. Massive vaccination is beneficial for suppressing the overall epidemic prevalence at the population level, but it also intensifies selective pressure favouring the emergence of vaccine-resistant viral strains^50^,^51^,^52^. In our data, there were 21 subjects who became sick with flu despite receiving flu shots earlier, confirming that the vaccines are not completely effective at containing novel strains^53^. But perhaps more importantly, they may exacerbate fears of vaccines since they provide the friends of the affected with first-hand evidence of vaccination failure that may spread from person to person and have a large impact on future vaccination rates.

Finally, the approach described here might also be used to study the interplay between the competitive diffusion of diverse outcomes – such as the epidemics of smoking cessation and obesity^24^,^27^ or the competitive spread of product purchases. A wide variety of human behaviors spread from person to person, but, so far, these have been primarily studied in isolation, one behavior at a time. We may find, as we do here, that the interaction of two or more contagions plays a critical role in the course of their spread and the resulting implications for public health interventions or marketing. It should be possible to build on the model presented here to develop further models of dueling contagions to improve our understanding of, and interventions regarding, human health.

## Additional Information

**How to cite this article:** Fu, F. *et al*. Dueling biological and social contagions. *Sci. Rep.* **7**, 43634; doi: 10.1038/srep43634 (2017).

**Publisher's note:** Springer Nature remains neutral with regard to jurisdictional claims in published maps and institutional affiliations.

## Supplementary Material

Supplementary Information

## Figures and Tables

**Table 1:** Comparative results of parameter estimates for candidate models.

Model parameters	Full model (0 < *a* < 1)	‘Rational response’ model (*a* = 0)	‘Social contagion’ model (*a* = 1)	No vaccination (*ω* = 0)
Transmission rate, *β*	0.1715 ± 0.0054	0.1421 ± 0.0032	0.1726 ± 0.0030	0.2708 ± 0.0089
Recovery rate, *γ*	0.1094 ± 0.0053	0.08022 ± 0.0029	0.1105 ± 0.0028	0.2624 ± 0.0104
Network responsiveness, *ω*	0.7028 ± 0.1955	4.4931 ± 0.0819	0.1948 ± 0.0113	—
Weight of peer influence, *a*	0.2435 ± 0.0801	—	—	—
Best estimated *R*_0_ = *β*/*γ*	1.564 ± 0.090	1.771 ± 0.075	1.562 ± 0.048	1.032 ± 0.053
RSS	**0**.**01485**	0.02506	0.01500	0.04418
AIC_*c*_ score	**−1605**.**0**	−1483.6	−1604.7	−960.16

Using the model selection approach, our aim is to tell whether vaccination decisions of individuals are driven by the epidemiological factor (the ‘rational response’ model with fixed *a* = 0), or by the social factor (the ‘social contagion’ model with fixed *a* = 1), or by both (the full model with both factors present and being weighted by the parameter 0 < *a* < 1). We also fit the epidemiological parameters, *β* and *γ*, using a pure disease transmission model with *ω* = 0: neglecting the responsiveness to either vaccination or infection results in a significant underestimate of *R*_0_. The full model gives the best fitting results with the smallest RSS and AIC_*c*_, whereas the ‘rational response’ model overestimates the disease severity (*R*_0_) and the time scale governing the increase in vaccine uptake (*ω*), and the ‘social contagion’ model underestimates *ω*. We also fit the data to the network-specific dueling contagion model (eqs 1, 2, 3, 4), and obtain similar results albeit with *R*_0_ and network responsiveness *ω* being overestimated (Figs S1 and S2), partly due to the sparseness of the mapped social network.
