# Supplementary material for: Dueling biological and social contagions
Source: Sci Rep. 2017 Mar 2;7:43634. doi: 10.1038/srep43634 (PMC5333634; doi:10.1038/srep43634)
Supplement: Supplementary Information [file srep43634-s1.pdf]

Supplementary Information for

Dueling biological and social contagions

Feng Fu[1,2], Nicholas A. Christakis[3,4,5], James H. Fowler[6,7,*]

[1] Department of Mathematics, Dartmouth College, Hanover, NH 03755, USA

[2] Department of Biomedical Data Science, Geisel School of Medicine at Dartmouth, Lebanon, NH 03756, USA

[3] Department of Medicine, Yale University, New Haven, Connecticut 06520, USA

[4] Department of Sociology, Yale University, New Haven, Connecticut 06520, USA

[5] Yale Institute of Network Science, Yale University, PO Box 208263, New Haven, Connecticut 06520, USA

[6] Division of Medical Genetics, University of California, San Diego, La Jolla, CA 92093, USA

[7] Department of Political Science, University of California, San Diego, La Jolla, CA 92093, USA

[*] Correspondence and requests for materials should be addressed to J.H.F. (email: fowler@ucsd.edu)

Contents

I. Data 3

II. Estimation method 5

III. Model selection criterion 6

IV. Network-specific dueling contagion model 7

V. Parameter estimates using self-reported flu data 10

VI. Time-scale separation analysis 10

References 13

I. DATA

We enrolled a total of 744 undergraduate students from Harvard College, discerned their friendship ties, and tracked whether they had the flu beginning on September 1, 2009, from the start of the new academic year, to December 31, 2009. Beginning on October 23, 2009, we approached 1,300 randomly selected Harvard College students (out of 6,650); we waited until a few weeks of the new school year had passed in order to be able to obtain current friendship information. Of these 1,300 students, 396 (30%) agreed to participate. All of these students were in turn asked to nominate up to three friends, and a total of 1,018 friends were nominated (average of 2.6 friends per nominator). This yielded 950 unique individuals to whom we sent the same invitation as the initial group. Of these, 425 (45%) agreed to participate. However, 77 of these 950 subjects were themselves members of the original, randomly selected group and hence were already participants. Thus, the sample size after the enrollment of the random group and the friend group was 744.

Nominated friends were sent the same survey as their nominators; hence, the original 425 friends also nominated 1,180 of their own friends (average of 2.8 friends per nominator), yielding 1,004 further, unique individuals. Although we did not send surveys to these friends of friends, 303 (30%) were themselves already enrolled either in the friends group or in the initial randomly selected group.

After giving informed consent, all subjects completed a brief background questionnaire soliciting demographic information, and flu and vaccination status since September 1, 2009. We obtained basic administrative data from the Harvard College registrar, such as sex and class of enrollment and tracked cases of formally diagnosed influenza among the students in our sample as recorded by University Health Services (UHS) beginning on September 1, 2009 through December 31, 2009. Presenting to the health service indicates a more severe level of symptomatology, of course, and so we do not expect the same overall prevalence using this diagnostic standard as with self-reported flu discussed below. However, UHS data offer the advantage of allowing us to obtain

information about flu symptoms as assessed by medical staff. A total of 627 of the 744 students (84%) who agreed to participate in the survey portion of our study also gave written permission for us to obtain their health records. Finally, 7 students reported being diagnosed with flu by medical staff at facilities other than UHS (in response to survey questions asked of all students), so we include these in the data as well.

Notably, we do not expect cases of flu to meaningfully alter the social networks and friendship patterns of Harvard undergraduates, let alone over a two-month period. And, we assume that the friendship network of Harvard students in our sample did not change meaningfully over the period September to December. That is, we treat the network as static over this time interval.

Beginning on October 23, 2009, we also collected self-reported flu symptom information from participants via email twice weekly (on Mondays and Thursdays), continuing until December 31, 2009. The enrolled students were queried about whether they had had a fever or flu symptoms since our last email contact, and there was very little missing data (47% of the subjects completed all of the biweekly surveys, and 90% missed no more than two of the surveys). Students were deemed to have a case of flu (whether seasonal or the H1N1 variety) if they report having a fever of greater than 100 F (37.8 C) and at least two of the following symptoms: sore throat; cough; stuffy or runny nose; body aches; headache; chills; or fatigue.

Hence, we had two measures of flu incidence. The medical-staff standard was a formal diagnosis by a health professional and typically reflected more severe symptoms. The self-reported standard captured cases that did not come to formal medical attention. As expected, the cumulative incidence of the latter was approximately four times the former (32% versus 8%) by the time of cessation of follow-up on December 31, 2009. We checked the sensitivity of our findings by using this self-reported measure of flu (see below).

As part of the foregoing biweekly follow-up, and to supplement the UHS vaccination records, we also ascertained whether the students reported having been vaccinated (with

seasonal flu vaccine or H1N1 vaccine or both) at places other than (and including) UHS.

For analyses using the network data we included 750 pairs of directed friendships among the participants (there were 158 mutual ties and 592 pairs of undirected friendships). The network degree of each subject is defined as the number of undirected friendships he/she has in this social network. Data collection and analysis was approved by the Harvard IRB committee.

II. ESTIMATION METHOD

We use the method of least squares to estimate the model parameters. We choose as the starting point the day when the very first incidences of both vaccination and infection take place. Our goal is to find parameter estimates that minimize the deviation between the predicted and the real trajectories of the temporal incidence of infected and vaccinated individuals over the time course under consideration. Denote by $\mathbf{q}^* = [\mathbf{q}_R{*}^T, \mathbf{q}_V{*}^T]^T$ the real data and by $\mathbf{q}(\mathbf{b}) = [\mathbf{q}_R^T, \mathbf{q}_V^T]^T$ the model predicted data. We need to find the parameters $\mathbf{b} = [\beta, \gamma, \omega, a]$ that minimize $e(\mathbf{b}) = ||\mathbf{q}^* - \mathbf{q}(\mathbf{b})||$, where $|| \cdot ||$ denotes the 2-norm. We constrain the search of the parameter space within the simplex $[0, 1] \times [0, 1] \times [0, 1] \times [0, 1]$.

We use simulated annealing to handle the presence of local minima in our parameter optimization search. We run the iteration $n = 10^6$ times, starting with a guess of the parameter values $\mathbf{b}_0$. The temperature T_i of each iteration is chosen to be $((n-i)/n)^4$. For each iteration i, we randomly choose a new sets of parameters $\mathbf{b}_1$ drawn from the close neighbourhood of $\mathbf{b}_0$ (using the Gaussian deviation $N(0, 0.01)$). If $e(\mathbf{b}_1) < e(\mathbf{b}_0)$, we set $\mathbf{b}_0 = \mathbf{b}_1$; otherwise with probability $\exp[(e(\mathbf{b}_0) - e(\mathbf{b}_1))/T_i]$, set $\mathbf{b}_0 = \mathbf{b}_1$. Throughout this search process, we obtain the best fitting parameters $\mathbf{b}$. The residual standard error is $\sigma_e = e(\mathbf{b})/\sqrt{k - m}$, where k is the number of observations and m the number of parameters. Denote by $\mathbf{J}$ the Jacobian matrix, where J_{ij} is given by

$$J_{ij} = \frac{\partial(q_i^* - q_i(\mathbf{b}))}{\partial b_j} = -\frac{\partial q_i(\mathbf{b})}{\partial b_j} \quad (1)$$

The Hessian matrix can be approximated by $\mathbf{H} \approx \mathbf{J}^T\mathbf{J}$. Accordingly the standard error of the parameter estimation b_i can be approximated by

$$\sigma_i = \sigma_e \sqrt{\mathbf{H}_{ii}^{-1}}. \quad (2)$$

III. MODEL SELECTION CRITERION

Following previous practice [1–3], we use the estimation method above to fit our data to four candidate models based on how individuals make vaccination decisions: (i) the full model (with parameter a) where individuals vaccinate in response to both social influence and disease prevalence, (ii) the 'rational response' model (with $a = 0$) where individuals base their vaccination decisions solely on the disease prevalence, (iii) the 'social contagion' model (with $a = 1$) where individuals vaccinate only when others have already done so, and (iv) the 'no vaccination' model that assumes vaccination does not play a role in the course of the epidemic. We use the modified Akaike information criterion (AIC_c) for model selection:

$$\mathrm{AIC}_c = -2\log(L_{max}) + 2m + \frac{2m(m+1)}{n-m-1}, \quad (3)$$

where the maximum likelihood estimator $L_{max} = (2\pi e \mathrm{RSS}/n)^{-n/2}$ (RSS is the residual sum of squares), m is the number of fitted model parameters, and n is the number of data points. The value of AIC_c indicates the goodness of fit and the desired model is the one with the lowest value of AIC_c.

Using the full model, the best estimated parameters are found to be $\beta_0 = 0.1715 \pm 0.0054\,\mathrm{day}^{-1}$, $\gamma_0 = 0.1094 \pm 0.0053\,\mathrm{day}^{-1}$, $\omega_0 = 0.7028 \pm 0.1955\,\mathrm{day}^{-1}$, and $a_0 = 0.2435 \pm 0.0801$. The residual standard error is 0.123. These estimates suggest a basic reproductive ratio of $R_0 \approx 1.56$. For the candidate model with fixed $a = 0$, the parameter search range for ω is set as $[0, 10]$. For network models, the effective $R_0 = \beta/\gamma\langle k^2\rangle/\langle k\rangle^2$, where the factor $\langle k^2\rangle/\langle k\rangle^2$ accounts for the effects of network heterogeneity on disease transmission (for homogeneous populations, it reduces to β/γ)[4, 5]. Parameter estimates for other candidate models give poorer fitting results and can be found in Table 1

of the main text. Furthermore, we repeated the same fitting procedure but using all available information regarding flu infections (i.e., self-reported flu cases[6]) and obtained somewhat larger estimates of the epidemiological parameters because of the higher incidence of self-reported flu (Table S1), but these additional results confirm that social contagion is a key determinant of vaccination behaviour.

IV. NETWORK-SPECIFIC DUELING CONTAGION MODEL

Aside from the "coarse-grained" results reported in Figs. 3 and 4 in the main text, we also fit the data to the network-specific dueling contagion model (Eqs. 1-4 in the main text). Because the mapped social network is very sparse and consists of a few components, we choose to focus only on its largest connected component (76 individuals) and use it to represent both the biological contagion network and the social contagion network. We report the best parameter estimates in the last column of Table S1. For comparison, we also plot similar figures corresponding to Figs. 3 and 4 in the main text (Figs. S1 and S2). We confirm that these results for the network-specific dueling contagion model are consistent with those reported in the main text: the final epidemic size could be mitigated approximately by half if the spread of vaccination behaviour was twice as fast or was driven solely by social contagion. Despite the intriguing one-humped curve in Fig. S2a (cf. Fig. 4a), the spread of flu is still most suppressed when individuals' vaccinations are influenced only by social contagion (a = 1, Fig. S2c).

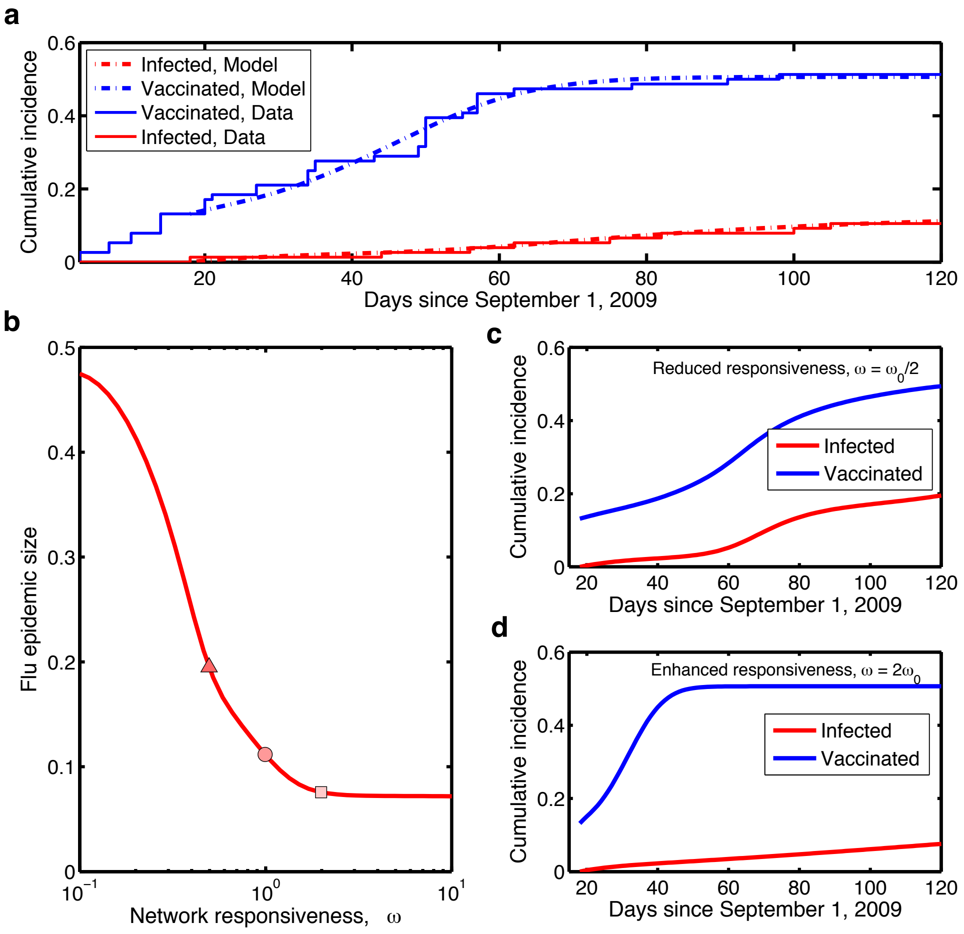

FIG. S1: Fitting results using the network-specific dueling contagion model. (**a**) Shown are the real data about the aggregate levels in the largest connected component of the mapped social network (solid) and the best-fitting curves (dashed) using a simple dueling contagion network model (Eqs. 1-4 in the main text). Panel (**b**) shows the dependence of the epidemic size (t = 120) on the network responsiveness, ω, for other model parameters fixed with the estimated values. The circle marks the estimated value of ω_0. (**c**) and (**d**) Plotted are the predictions of population aggregate behaviors, based on the network-specific SIRV model of the dueling contagion processes, for a smaller $\omega = \omega_0/2$ (the triangle in panel **b**) and for a larger $\omega = 2\omega_0$ (the square in panel **b**), respectively.

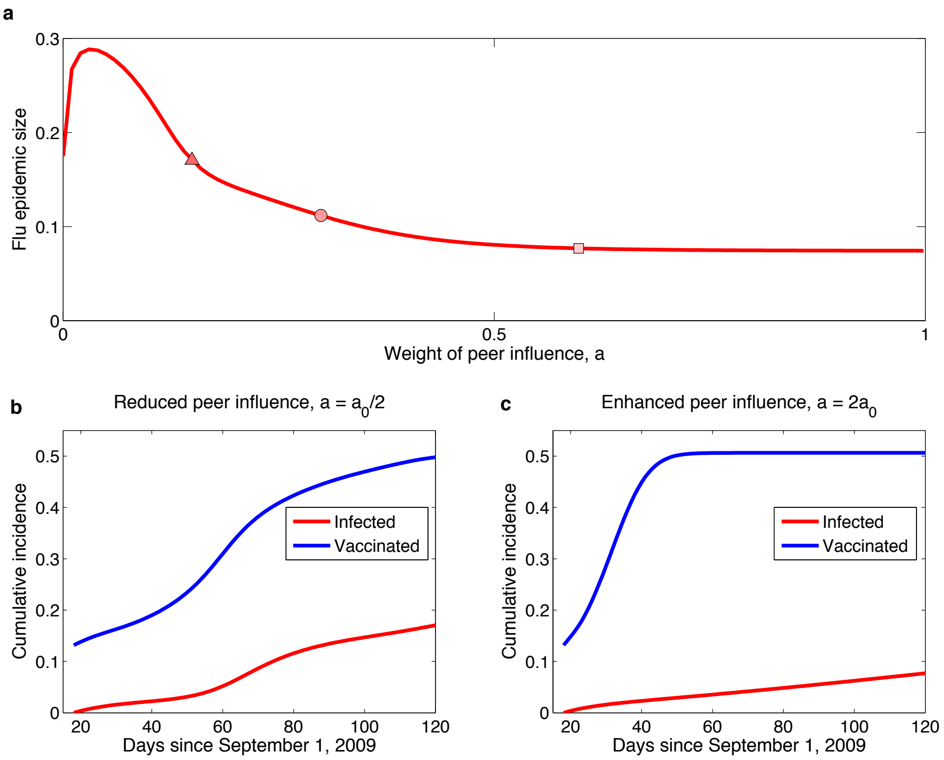

FIG. S2: Quantifying the impact of social contagion on health outcomes using the network-specific dueling contagion model. Panel (**a**) depicts the final epidemic size ($t = 120$) as a function of the parameter a describing the extent of the role that social contagion, in comparison to the risk of infection, plays in an individual's vaccination decision. The circle marks the estimated value of $a_0 \approx 0.30$ inferred from our real data. Panels (**b**) and (**c**) plot the aggregate levels of vaccination and infection, predicted by our network-specific dueling contagion model, with halving ($a = a_0/2$, the triangle in panel **a**) versus doubling ($a = 2a_0$, the square in panel **a**) the relative effect of peer influence on vaccinating decisions of individuals.

V. PARAMETER ESTIMATES USING SELF-REPORTED FLU DATA

We performed similar parameter estimates as in the main text but using all available information regarding flu infections (through self-report of subjects). Not surprisingly, the total number of self-reported flu illness far exceeds that of diagnosed flu, that is, 237 versus 57. As a result, self-reported flu data gives a much higher estimation of the epidemiological parameters ($R_0 \sim 2.9$). It is worth noting that, albeit with much elevated flu incidence (namely, higher perceived risk of infection) in this situation, the 'rational response' model does not fit better than the 'social contagion' and the full model. In line with our conclusion in the main text, this additional result suggests that social contagion is a key determinant of vaccination. Detailed fitting results using self-reported flu data can be found in Table S1.

VI. TIME-SCALE SEPARATION ANALYSIS

We describe the dueling contagion processes of vaccination and infection based on the following aggregate fractions:

$$\frac{d\rho_S}{dt} = -\omega(\kappa - \rho_V)[a\rho_V + (1-a)\rho_I] - \beta\rho_S\rho_I, \tag{4}$$

$$\frac{d\rho_I}{dt} = \beta\rho_S\rho_I - \gamma\rho_I, \tag{5}$$

$$\frac{d\rho_R}{dt} = \gamma\rho_I, \tag{6}$$

$$\frac{d\rho_V}{dt} = \omega(\kappa - \rho_V)[a\rho_V + (1-a)\rho_I]. \tag{7}$$

We find no closed-form solutions to the ordinary differential equations above, but we can obtain analytical approximations using the time-scale separation technique for extreme values of ω.

Specifically, for $\omega \to 0$ (when individuals show very little or no responsiveness to either vaccination or infection), the spread of vaccination occurs much more slowly, compared to the spread of infection. Thus, the dueling contagion dynamics will con-

TABLE S1: Parameter estimates based on self-reported flu data, as similarly done in Tab 1 in the main text.

	Self-reported flu data					Diagnosed flu data
Model parameters	No vaccination ($\omega = 0$)	'Rational response' model ($a = 0$)	'Social contagion' model ($a = 1$)	Full model ($0 < a < 1$)	Network model ($0 < a < 1$)	Network model ($0 < a < 1$)
Transmission rate, β	0.2242 ± 0.0036	0.1603 ± 0.0024	0.1605 ± 0.0017	0.1598 ± 0.0019	0.9995 ± 0.0192	0.3738 ± 0.0143
Recovery rate, γ	0.1755 ± 0.0036	0.0553 ± 0.0023	0.0550 ± 0.0017	0.0545 ± 0.0018	0.3027 ± 0.0110	0.1485 ± 0.0097
Network responsiveness, ω	-	1.6187 ± 0.0425	0.1943 ± 0.0018	0.2224 ± 0.0970	0.9648 ± 0.6100	0.9939 ± 0.7369
Weight of peer influence, a	-	-	-	0.8583 ± 0.4269	0.2832 ± 0.1896	0.2990 ± 0.2291
Best estimated $R_0 = \beta/\gamma$	1.28 ± 0.03	2.90 ± 0.13	2.92 ± 0.09	2.93 ± 0.10	3.95 ± 0.16	3.01 ± 0.23
RSS	0.1757	0.0707	0.0404	0.0149	0.0509	0.0424
AIC_c score	−634.4	−1238.9	−1371.1	−1605.0	−876.0	−1155.8

verge to the slow dynamic of vaccination, $\frac{d\rho_V}{dt} = \omega(\kappa - \rho_V)[a\rho_V + (1-a)\rho_I]$, following the fast dynamic of infection in which the spread of vaccination is decoupled and has almost no effects on the disease transmission:

$$\frac{d\rho_S}{dt} = -\beta\rho_S\rho_I, \tag{8}$$

$$\frac{d\rho_I}{dt} = \beta\rho_S\rho_I - \gamma\rho_I, \tag{9}$$

$$\frac{d\rho_R}{dt} = \gamma\rho_I. \tag{10}$$

$$\tag{11}$$

It yields:

$$\frac{d\rho_S}{\rho_S} = -R_0 d\rho_R. \tag{12}$$

Integrating the above equation, we get:

$$\ln\rho_S(\infty) - \ln\rho_S(0) = -R_0(\rho_R(\infty) - \rho_R(0)). \tag{13}$$

Using the initial condition, $\rho_S(0) \sim 1$ and $\rho_R(0) \sim 0$, and $\rho_S(\infty) = 1 - \rho_R(\infty)$ at the end of the epidemic spreading, we obtain the transcendental equation below, which determines the final epidemic size, ρ_R:

$$\rho_R = 1 - \exp[-R_0\rho_R]. \tag{14}$$

For $R_0 = 1.56$, ρ_R is approximately 0.61.

On the other limit, for sufficiently large values of ω (when the population shows high levels of responsiveness), the spread of vaccination behavior is a fast dynamic and rapidly converges to the equilibrium level κ, while the spread of flu infection is a slow dynamic. Therefore, the epidemic spreading dynamic recovers to the classic SIR model with the pre-emptive vaccination level κ. In our study, the equilibrium level of vaccination is 0.415, which exceeds the herd immunity threshold, $1 - 1/R_0$ (0.35), so the disease cannot persist in the population, $\rho_R \sim 0$, in this scenario.

For intermediate values of ω, the dueling contagion dynamics exhibit strong mutual interdependence (i.e., no time-scale separation): the epidemic size ρ_R monotonically

decreases with the increasing network responsiveness, ω, leading to a transition from high numbers of infection to rare infections, as shown in Fig. 3b in the main text.

---

[1] Bauch, C.T., & Bhattacharyya, S. (2012). Evolutionary game theory and social learning can determine how vaccine scares unfold. PLoS Computational Biology 8(4): e1002452.

[2] Burnham, K. P., & Anderson, D. R. (2004). Multimodel inference understanding AIC and BIC in model selection. Sociological Methods & Research 33(2): 261-304.

[3] Oraby, T., Thampi, V., & Bauch, C. T. (2014). The influence of social norms on the dynamics of vaccinating behaviour for paediatric infectious diseases. Proceedings of the Royal Society B: Biological Sciences 281(1780): 20133172.

[4] Anderson, R. M., May, R. M., & Anderson, B. (1992). Infectious diseases of humans: dynamics and control (Vol. 28). Oxford: Oxford university press.

[5] May, R. M., & Lloyd, A. L. (2001). Infection dynamics on scale-free networks. Physical Review E 64(6): 066112.

[6] Christakis, N.A., & Fowler, J.H. (2010). Social network sensors for early detection of contagious outbreaks. PLoS ONE 5: e12948.
